# Impact of tunnelling on piles in Parisian subsoil: dataset of in-situ measurements in the ground and on three instrumented piles

**DOI:** 10.1016/j.dib.2023.108971

**Published:** 2023-02-11

**Authors:** Nicolas BERTHOZ, Denis BRANQUE, Agathe MICHALSKI, Wassim MOHAMAD, Emmanuel BOURGEOIS, Alain LE KOUBY, Fabien SZYMKIEWICZ, Antoine RALLU

**Affiliations:** 1 French Centre for Tunnel Studies (CETU), Bron, France; 2 University of Lyon, ENTPE, LTDS - UMR CNRS 5513, Vaulx-en-Velin, France; 3 Université Gustave Eiffel (UGE), Marne-la-Vallée, France

**Keywords:** Surface settlements, inclinometers, extensometers, normal force in piles, TBM, soft ground, pile interaction

## Abstract

A major full-scale experiment (the TULIP - Tunneling and Limitations on the Impact on Piles - research project) was conducted in 2020 on Line 16 of the Grand Paris Express project (France). Its objective was to analyze the tunnel boring machine-soil-pile interactions occurring during tunnel excavation near piled structures in the context of the geology of the Paris basin. This data paper summarizes the main measurements made during this experiment, namely: (i) the horizontal and vertical displacements measured in the ground, on the surface and in the thickness of the cover, (ii) the settlements of the pile heads, and the variations of the normal forces in the pile depth. These data, interpreted in two articles cited in the references, may be of interest for the calibration of analytical and numerical models dedicated to the estimation of the impact of TBM excavation on neighboring constructions, in particular those based on piles.

**Specifications Table**

**Table:** 

Subject	Civil and Structural Engineering

Specific subject area	Displacements and variations of normal forces induced by tunnelling on the ground and on piles
Type of data	Table Figure
How the data were acquired	The experimental set-up is composed of: topographic prisms on the ground surface and on piles heads, inclinometers and extensometers into the ground, and vibrating-wire strain gauges into the piles
Data format	Raw
Description of data collection	The data were acquired during the loading of the piles, then during the passage of the tunnel boring machine in the experimental set-up, on July 2020. The measurements were taken as a function of time and then cross-referenced with the progress of the TBM. The data files presented here correspond to the displacements and forces as a function of the distance to the working face.
Data source location	- Institution: measurements made on line 16 of Grand Paris Express (precise location below), and analyzed in University of Lyon, French Centre for Tunnel Studies, and Université Gustave Eiffel - City/Town/Region: Aulnay-sous-Bois - Country: France - Latitude and longitude: 48.955147, 2.481519
Data accessibility	The raw data are hosted in the following link: https://mycore.core-cloud.net/index.php/s/tOWOrhHBkvVhxOz
Related research article	W. Mohamad, E. Bourgeois, A. Le Kouby, F. Szymkiewicz, A. Michalski, D. Branque, N. Berthoz, L. Soyez, C. Kreziak (2022), Full scale study of pile response to EPBS tunnelling on a Grand Paris Express site, Tunnelling and Underground Space Technology, 124, https://doi.org/10.1016/j.tust.2022.104492

## Value of the Data

• A full-scale in-situ experiment, rare on an international scale, was carried out to better understand TBM/ground/pile interactions.
• The data presented may be useful in the context of comparisons of the kinematics of the ground and the piles observed on this site, with other data acquired on other projects.
• These data can also be used as a support for the development and calibration of numerical models dedicated to TBM/ground/pile interactions.
• These data can be useful to researchers or engineers in design offices and underground works companies.

## 1 Objective

Reducing the mechanical impact on neighbouring buildings is now a major issue for underground projects in urban areas. This issue has been widely studied in the laboratory in the case of tunnelling close to buildings founded on piles. Nevertheless, only a few full scale experiments have been carried out. The TULIP research project (2018-2023) was thus created to provide new elements for understanding the mechanisms involved and to contribute to the improvement of calculation methods for design on this subject. The core of the project consists of a full-scale experiment and numerical modelling, whose essential data are presented here, and interpreted in [1] and [2].

## 2 Data Description

The following raw data files (format .csv, separated by semicolon) are available on the link given above. The associated graphics are available in [1] and [2].

*Concerning the progress of the TBM:*

- **Progress.csv:** This file contains the TBM progress as a function of the time. The second column correspond to the position of the cutterhead. SMRN is located on metric point 813.7, SMRS is located on metric point 853.0, and the two pore pressure cells are located on metric point 851.0.

*Concerning the ground surface displacements:*

**Fig. 1:**
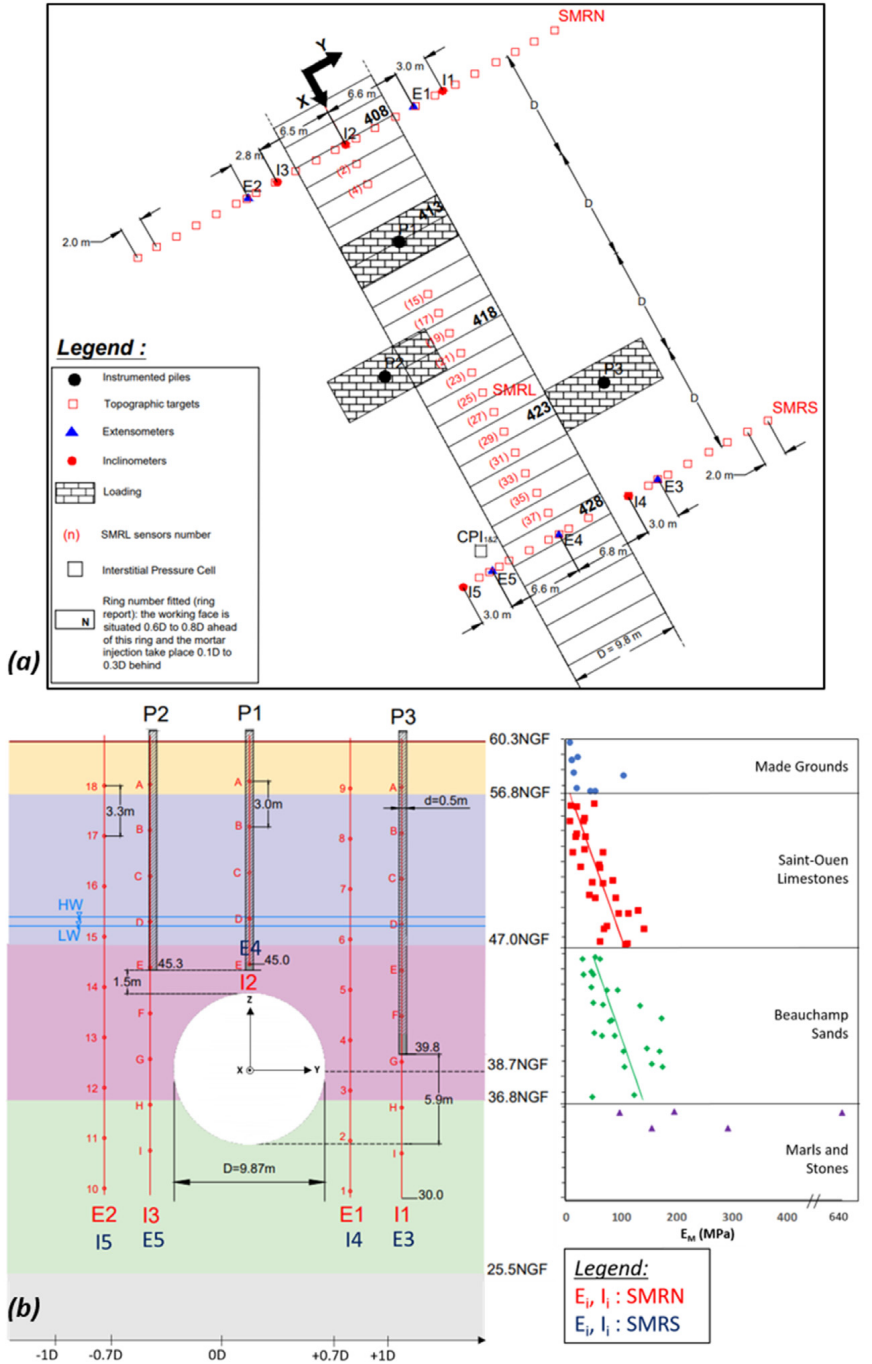
(a) Plan view of the site showing surface positions of instrumentation elements and instrumented piles, (b) Cross-section showing the positions of the piles with respect to the tunnel, and Menard pressuremeter modulus.
- **SMRN**-**Uz.csv:** this file contains the evolution of the vertical displacement (in mm) of different topographic prism on the transverse SMRN line (Fig. 1a) in function of the transverse distance Y_st_ between the prism and the tunnel axis. The first column correspond to the values of Y_st_. The other columns of the table correspond to the vertical displacements (in mm) for different position of the section related to the face (distance X_st_).
- **SMRS-Uz.csv:** this file contains similar data than SMRN-Uz.csv, but for the SMRS section.
- **SMRN-Uy.csv:** this file contains similar data than SMRN-Uz.csv, but for horizontal transverse displacements.
- **SMRS-Uy.csv:** this file contains similar data than SMRS-Uz.csv, but for horizontal transverse displacements.

- **SMRL-Uz.csv**: this file contains the evolution of the vertical displacement (in mm) of different topographic prism on the longitudinal SMRL line (Fig. 1a) in function of the horizontal distance X_st_ between the prism and the TBM cutterhead (divided by the cutterhead diameter). Each couple of column correspond to a prism “SMRL_i_” of the SMRL line. The index i corresponds to the longitudinal distance between the prism considered and the line SMRN.

*Concerning the ground subsurface displacements:*

- **ExtensoE1-Uz.csv:** this file contains the vertical displacements (in mm) measured for each anchor in function of the depth z (first column of the raw data). The different columns correspond to different position of the extensometer related to the face (distance X_st_).
- **ExtensoE2-Uz.csv to ExtensoE5-Uz.csv:** this file contains similar data than ExtensoE1-Uz.csv but for the extensometers E2 to E5.
- **InclinoI1-Uy.csv:** this file contains the horizontal transverse displacements (in mm) measured for each anchor in function of the depth (first column of the raw data). The different columns correspond to different position of the inclinometer related to the face (distance X_st_).
- **InclinoI2-Uy.csv to InclinoI5-Uy.csv:** this file contains similar data than InclinoI1-Uy.csv but for the inclinometers I2, I3, I4 and I5.
- **InclinoI1-Ux.csv:** this file contains the horizontal longitudinal displacements (in mm) measured for each anchor in function of the depth (first column of the raw data). The different columns correspond to different position of the inclinometer related to the face (distance X_st_).
- **InclinoI2-Ux.csv to InclinoI5-Ux.csv:** this file contains similar data than InclinoI1-Ux.csv but for the inclinometers I2, I3, I4 and I5.

*Concerning the pore pressure in the ground:*

- **CPI.csv:** this file contains the evolution of the pore pressure (in kPa) of the two pore pressure cells (CPI1 and CPI2) in function of the time.

*Concerning the response of the piles to loading and tunnelling:*

- **Piles-loading.csv:** this file contains six columns. Columns 1, 3, 5 corresponds to the loads (in kN) applied of the head of piles P1, P2 and P3 respectively. Columns 2, 4, 6 corresponds to the settlements (in mm) of these heads of piles.
- **PileP1-Normal-force.csv:** this file contains for the pile P1, the distribution of the normal force in the depth (z in m) of the pile for different dimensionless distance X_pt_ between the pile and the cutterhead. Each column corresponds to a value of X_pt_.
- **PileP2-Normal-force.csv and PileP3-Normal-force.csv:** this file contains similar data than PileP1-Normal-force.csv for the pile P2 and P3.
- **Piles-Uz.csv:** this file contains the vertical displacements (in mm) of the head of the piles, in function of the distance (dimensionless because divided by the cutterhead diameter) X_pt_ between the piles and the cutterhead.

## 3 Experimental Design, Materials and Methods

*Experimental set-up:*

 A plan view and a cross-section view of the full-scale experimentation are presented in Fig. 1. The tunnel has an outer diameter of 9.5 m (excavated diameter D = 9.87 m) and the depth of the axis is around 21 m at the experimental site. The stratigraphy at the site reveals sub-horizontal formations: made grounds (MG), marly Saint-Ouen limestone (SOL), fine clayey Beauchamp Sands (BS) and whitish indurated and fragmented marly limestones (MS). Informations about the hydrogeological and geomechanical characteristics are available in [1] and [2].
 Three reinforced concrete piles named P1, P2 and P3, with an outer diameter of 500 mm have been realized. As shown in Fig. 1b, the transverse distance between the axis of each pile and the axis of the tunnel, as the depth of each pile, are different in order to belong to different influence zones identified in the literature. The piles were loaded, using hydraulic jacks and kentledge reaction platforms made of steel beams (without reaction piles), up to 2100 kN, which corresponds to approximately 54% of the estimated ultimate bearing capacity of P1 and P2 and 33% of P3.

*Ground instrumentation:*

 The ground instrumentation includes measurements of:
  (i) Displacements in the three directions (u_X_, u_Y_, u_Z_) of topographic prisms placed at the surface followed by three high accuracy measuring stations (T1, T2, T3). Taking into account the viewing distances and local weather conditions, successive measurements showed that the accuracy of the measurements is in the order of +/- 0.2 mm. As the stations were placed in the geotechnical influence zone of the tunnel, some “reference” targets (outside the geotechnical influence zone of the TBM) were measured at each measurement cycle to recalibrate the displacement of the stations themselves. The displacement measurements of all three stations were corrected for atmospheric conditions. The correction took into account air pressure, air temperature and humidity.
  (ii) Vertical subsurface ground displacements using five multipoint borehole extensometers (E1 to E5). The extensometers used had nine anchors spaced 3.3 m apart. The relative displacement of each anchor with respect to the head of each extensometer was measured with an accuracy of +/-0.1 mm. The head displacements of the extensometers were followed by the total stations. The absolute displacements of the anchors could thus be deduced, with an accuracy of +/-0.2 mm.
  (iii) Horizontal subsurface ground displacements in the longitudinal (u_X_) and transverse direction (u_Y_) with five automatic inclinometers (I1 to I5). Each inclinometer tube comprised a chain of nine sensors (spaced 3 m apart) whose rotation relative to the vertical direction was measured over time. The angular accuracy of the measurements for each sensor was equal to 0.5°. The horizontal displacements were deduced from the angular variations by integration, considering a known displacement point. For the deepest inclinometers (I1, I3, I4, I5), the displacement of the base of the inclinometer could be considered to be zero due to the rigidity of the Marls and Stones (MS). The topographic monitoring of the head of the inclinometers on the surface made it possible to confirm the displacements calculated by integration of the rotations from the base. For the I2 inclinometer located above the tunnel axis, the absolute horizontal displacements at depth were calculated from the horizontal surface displacement deduced from the topographic measurements.
  (iv) Pore pressure with two pore pressure sensors (CPI1 and CPI2). Each sensor was placed in a dedicated borehole, surrounded by sand (drainage material) with a bentonite plug sealing the borehole (closed piezometer). The accuracy of these pore pressure cells is ±2 kPa.

The surface and internal displacement sensors are positioned along three measurement lines: the first one (SMRN) is located 1.D before the P1-pile on the North side, the second one (SMRS) is located 1.D after the P3-pile on the South side, and the third one (SMRL) is in the longitudinal direction, above the tunnel axis, between the two cross-sections.

All field measurements were started one month before the arrival of the TBM in the TULIP experimental zone. This “dry run” period allowed the smooth operation of the device to be observed and the absence of natural displacements (not related to the tunnel work) to be verified. All the measurements were then acquired every 20 minutes throughout the TBM's passage through the experimental site and until the ground displacements were stabilized. The measurements processed concern the period from 03/06/2020 to 14/08/2020. The acquisition frequency of the pore pressure measurements was increased to one per second when the tunnel face was located within twenty meters (≈2 D) of the pore pressure sensors.

*Pile instrumentation:*

 The piles were instrumented with:
  (i) Vibrating-wire strain gauges to monitor strains during loading of the piles then tunnelling. The pile reinforcing cage comprised eight HA14 bars, uniformly distributed. Strain gauges were distributed at 90° intervals, attached to the cages by means of plastic cable ties, and with a vertical spacing of 2 m for P1 and P2 and 3 m for P3. The other four HA14 bars were equipped with optical fiber sensors that are not presented in the paper [2].
  (ii) Topographic prisms to get pile head displacements. The frequency and accuracy of these measurements are identical to those of the ground described previously.
  (iii) Load cells at the pile head to monitor applied load variations.

*TBM instrumentation:*

 The TBM used is an Earth-Pressure-Balanced Shield. It is equipped with the type of instrumentation available on current TBMs. In particular, twelve pressure sensors are located behind the cutterhead to measure the pressure of the excavated material on the walls of the working chamber. The total thrust force, the thrust force transiting through the shaft of the cutting wheel, the torque on the cutting wheel, the tail grout volume and pressure, the weight of excavated material on the conveyor belt are also monitored.
 The TBM parameters measured during the crossing of the experimental campaign are detailed in [1]. Just remember here that:
  (i) the face pressure in the axis was equal to 175±20 kPa,
  (ii) the force applied by the cutter head on the ground was equal to 7000 ± 2000 kN,
  (iii) no bentonite was injected along the shield,
  (iv) the injection pressure of the mortar behind the ring was equal to 200 ± 25 kPa (value in the tunnel crown).

## Ethics Statements

The present work did not involve the use of human subjects, animal experiments, or data collected from social media platforms.

## CRediT authorship contribution statement

**Nicolas BERTHOZ:** Conceptualization, Writing – original draft. **Denis BRANQUE:** Conceptualization, Writing – review & editing. **Agathe MICHALSKI:** Formal analysis, Visualization, Investigation. **Wassim MOHAMAD:** Formal analysis, Visualization, Investigation. **Emmanuel BOURGEOIS:** Conceptualization, Writing – review & editing. **Alain LE KOUBY:** Writing – review & editing. **Fabien SZYMKIEWICZ:** Writing – review & editing. **Antoine RALLU:** Visualization, Data curation.

## Declaration of Competing Interest

The authors declare that they have no known competing financial interests or personal relationships that could have appeared to influence the work reported in this paper.

## Data Availability

TULIP-Ground+piles (Original data) (TULIP).
